# Tetanus1Read before the Iowa State Veterinary Medical Association, at Des Moines, Sept. 9, 1895.

**Published:** 1896-01

**Authors:** S. H. Kingery

**Affiliations:** Creston, Iowa


### TETANUS.[1]

BY S. H. KINGERY,
CRESTON, IOWA.

THIS is a term applied to that infectious disease of the horse characterized by involuntary, painful, and continued or tonic spasm of more or less extensive groups of voluntary and involuntary muscles, these spasms during their continuance being marked by periods of exacerbation and of repose.

*Pathology, nature, and causation.* It has been the opinion of some very learned authors that tetanus depends, first, upon the excessively excited state of the nerve-tissue of the spinal cord induced by hyperæmia and morbid conditions of the bloodvessels, exudation, and disintegration resulting therefrom; secondly, that the spasms are the result of the persistent irritation of the peripheral nerves, by which the exalted excitability of the cord is aroused, and thus the cause which at first induced in the cord its morbid susceptibility to reflex action is subsequently the source of that irritation by which the reflex action is excited.

We cannot go back on the fact that it is an excited condition of the nervous system, and considered to be associated with a peculiar condition of the blood, produced undoubtedly by the ravages of the specific bacillus of tetanus. Thanks to some of the observers, as Rosenbach, Nicolaier, and Kitasato, the specific bacillus has been discovered. Carle and Rattone, in 1884, furnished the first proof of the communicability of the disease by inoculation of rabbits with pus from a wound in a case of human tetanus. Nicolaier, in 1885, found disseminated in all kinds of earthy matter bacilli which, introduced subcutaneously into mice, guinea-pigs, and rabbits, produced typical trismus and tetanus with fatal termination. Rosenbach, in 1886, demonstrated the tetanus bacillus for the first time in man. Thus was established the generic relation to the diseases of the bacillus of Nicolaier. The poison appears to be in the ptomaïns, a specific product of the tetanus bacillus. Kitasato observed that the filtrate, perfectly free from germs, produced the same tetanic effect. The ptomaïns, such as tetanin and tetanotoxin, have been extracted from the culture of this bacillus, and it is probable that most of the symptoms of irritation of the nervous system are due to the presence of these substances. The bacillus is a short rod with an enlargement at

[1] Read before the Iowa State Veterinary Medical Association, at Des Moines, Sept. 9, 1895.

one end, due to sporulation, which gives it the characteristic drum-stick shape. Although it is found in the dust of the street, it rarely finds an opportunity to grow in the living tissues, owing to its anaërobic properties, hence the rarity of the disease. The bacilli are found principally in tissue near the wound as well as in the internal organs and blood.

It occurs in all animals, but is seen most often in the horse. It occurs most often in hot weather, during the prevalence of thunder-storms, indicating a superabundant amount of electricity in the air. It becomes enzoötic in such cases. Horses are more subject to it in damp, filthy, basement stables. This and another reason—namely, the effect of strychnine—go to prove that the disease is associated with some abnormal condition of the blood. It would seem that some particular state of the system is a necessary precursor of this malady. That tetanus may be produced through the blood is shown by the results of the administration of strychnine, which imitates the tetanic symptoms in a very striking manner, so that you may at will develop the phenomena of tetanus in an animal by giving him strychnine or injecting it into his blood, but you cannot cause it by external injury.

Tetanus is of two kinds—traumatic and idiopathic. When the muscles of face and neck are chiefly affected, resulting in persistent closure of the jaws, which is characterized as lock-jaw, it is spoken of as trismus; when the muscles of one side of the body are affected it is known as pleurothotonus; when the dorsal and upper cervical muscles are involved it is known as opisthotonus; when the muscles of belly are affected it is then called emprosthotonus; when the muscles of the whole animal are disturbed it is then known as orthotonos.

The traumatic form follows the infliction of wounds in from two to twenty days, rarely as late as thirty-five days. It may follow any wound, slight or severe, or various operations, as castration, docking, etc., but mostly follows badly treated or neglected wounds; most commonly follows wounds caused by a nail in the foot, or the wound occasioned by castration. Punctured wounds generally offer the best opportunity for the growth of the bacillus, and if such wounds are inflicted on parts of the body naturally coming in contact with dust and dirt, as the legs and feet, or if foreign bodies covered with dust or dirt, containing the bacilli, are lodged in the tissues, the condition favorable for infection is obtained.

The idiopathic form arises from unseen causes: exposure to extremes of climatic influence and to fatigue, especially among animals with insufficient food and improper sanitary conditions; intestinal irritation, produced by indigestible material or parasites; metritis following parturition. It may follow any slight disease, even overdriving or overheating in hot weather.

*Symptomatology.* From the outset to the very termination the symptoms may be said to be diagnostic. Take a typical case. The first thing you see is slight trismus; grinding of the teeth; a drawn condition of the muscles at the angles of the mouth; increased flow of saliva secreted—may drop from mouth to the ground; the superior cervical muscles contracted, elevating the head and causing the nose to become protruded. On entering the stall you will notice unnatural excitability. The horse is nervous. There will be twitching of the muscles of the face; the eye will be drawn upward and backward into the orbit, showing the white of the eye and making it look smaller, and at the same time the membrana nictitans is drawn upward over the eye; the limbs are forcibly extended and kept widely apart; locomotion is difficult, and there is a peculiar straddling, stilty gait, with evident pain.

As the disease develops the aggravation of the symptoms is usually rapid. The spasms extend to all parts of the body; the muscles over the body become rigid and hard to the touch, especially in the cervical, dorsal, and gluteal regions; the tail is elevated and trembles, the nostrils dilated, and the ears stiff and pointed upward. The eye remains retracted in the orbit, and the membrana nictitans remains drawn upward over the eye. Respiration is accelerated; flanks tucked; there will be contraction of the abdominal muscles; ribs drawn tightly, diminishing the size of chest; the nose elevated and extended more; will sweat very freely. The whole nervous system is in a state of tonic spasm, but spasms are more or less intermittent; the relaxations are not very apparent while the exacerbations are. The horse in this condition is very sensitive to all noises and to the presence of other animals.

When the disease is fully developed and the jaws are locked there is inability to eat, and streams of ropy saliva hang from the mouth. The pulse at first is slightly hardened and also accelerated, but as the diseases progresses becomes very hard, small, and weak, and continues more so as the disease advances. The desire for food and water generally remains good, and bowels generally inactive. The horse persistently stands in bad cases. In mild cases they usually lie down, and get up alone. This is considered a favorable symptom. Sometimes, in cases not severe, horses will get up after the spasm has passed, and again they are unable to get up after once down. There is apparently terrible distress in the præcordium. Would describe it as of a painful, dragging nature, and no doubt dependent upon spasmodic contraction of the diaphragm. At this time excitement increases, and the animal dies from exhaustion, caused by tonic spasm of the muscles of the heart. Death takes place usually in from four to twenty days, or sooner even than four days; sometimes in forty-eight to sixty hours in very bad cases.

*Prognosis.* The idiopathic form usually terminates favorably. The traumatic, when occasionally of a mild character, and when all symptoms are not fully developed, and occupying in the passage through its various phases a rather lengthened period, has a favorable prognosis. In severe cases, which are characterized from the accession of the diagnostic symptoms of tonic muscular spasms to their full development by rapidly recurring exacerbations, febrile disturbance, high temperature, much restlessness and pain, the percentage of recoveries is exceedingly small, but still much depends upon the surroundings and nursing. If in these cases the patients live twenty days they generally recover, especially if the temperature of the body remains within normal limits. It must be remembered that an apparently favorable case sometimes takes a rapidly unfavorable turn and death ensues.

*Treatment.* Very many cases of tetanus we are called upon to treat are, from the outset, evidently hopeless. These rarely survive long enough to give any therapeutic agent an opportunity of acting upon the system. In all cases the first matter demanding attention is the hygienic treatment. It consists in placing the animal in a comfortable box-stall, with good ventilation, dry, dark, and perfectly quiet. It is best to keep the same attendant all the time; do not change. Keep away all visitors and spectators. Keep soft, sloppy mashes before him all the time. Would recommend placing the horse in a sling early. Clothe the animal according to the time of the year. Avoid all noisy demonstrations. Sudden flashing of a light in a previously darkened stable, hurriedly and roughly opening a door, a sudden attempt to take hold of the animal, or talking loudly, are sufficient to excite the animal and retard its recovery.

Medical Treatment: Unless the bowels are already in a lax state, a moderate dose of aloes should be given early. Antispasmodics and anodynes are indicated; dilute prussic acid, ext. belladonna, calabar bean, sulphate of morphine, chloral hydrate, the bromides, fluid-extract lobelia, ext. physostigmin, have all in turn been tried with varied results. The various remedies may be given with syringe per mouth, rectum, or hypodermatically, according to the severity of the case. The solid extract of belladonna is best smeared on the teeth and the horse allowed to suck it. Sometimes the medicine may be given in mashes, as appetite often remains good. If the case is one of traumatic tetanus, the wound ought to be carefully examined, and all foreign matter or animal tissue which, from necrosis, is acting as an irritant removed, then apply a hot, soft, linseed poultice, medicated with carbolic acid, so as to let the poultice come into contact with the wound. Dress the wound twice a day. Extract of belladonna is recommended to be smeared over the wound in whatever region it may be located.

I think electro-therapy ought to receive more attention among veterinarians than it does in the treatment of nervous diseases. For my own part, I can speak very highly of its application in the treatment of tetanus. It may be a matter of mere chance or luck, so-called; but, anyway, success seemed to follow its use. During the time elapsing between May 4 and 22, 1894, I had ten cases of traumatic tetanus, six occurring in one week and all following the operation of emasculation. Eight of the ten cases recovered. Five out of six cases treated by electricity recovered, and three out of four treated otherwise. Six cases were in the city, and four were several miles in the country. The six cases were all in the hospital, where treatment with the battery was comparatively easy. The machine being roughly constructed by a local electrician, and not being portable, I could not use it on cases in the country. These cases, with the exception of one, were very mild, and yielded readily to medicinal remedies. The six cases at the hospital were apparently aggravated. The battery used was a faradic battery similar in construction to a McIntosh twelve-cell faradic battery, with sponge electrodes; the current instead of coming directly from the battery-cells passes through an induction-coil.

I must confess that I am not very well versed in electrical appliances, or in knowledge of electricity, as the above was a matter of experiment with me; but thinking that it might prove beneficial to the profession at large, I bring it before you. Eight cells out of the twelve were sufficient for producing a current strong enough in the cases I treated, as they were under two years of age. I found by using more there would be spasmodic contraction of the group of muscles where the electrodes were applied; in one case sufficient to throw the animal down. Under the direct use of eight cells the horse at first showed signs of excitement, but after three or four applications, one day apart, would take the treatment with no visible excitement. After the second day could see improvement in various symptoms. Continued treatment in all cases eight to fourteen days, with complete recovery. I used general faradization. One electrode, usually the positive, is placed either at the base of the tail or in whatever region may suggest itself to the operator, and the other negative electrode moved over the body.

Another form of treatment is with tetanus antitoxin, which ought to be used more extensively than it is, being comparatively new as yet, but still not without its friends. Numerous cases have been cured in the human family by the aid of this remedy, principally in foreign countries. Only once has it been tried in America, and that at Amsterdam, N. Y., by Dr. C. F. Timmerman, in a man, nineteen years of age, having the disease, with complete recovery.

In a recent letter from Dr. Paul Gibier, of the Pasteur Institute, New York City, he informed me that he personally knows of several cases being cured by its use in the horse.

*Anatomical characters.* A casual examination reveals nothing wrong, but on a close examination by the aid of the microscope you will find congestion of the neurilemma (or covering of the nerves) leading to and from the wound, the bloodvessels of the spinal cord engorged, and usually effusion in the subarachnoid space. The blood, as well as the tissue in the immediate vicinity of the wound, will be found in some cases crowded with minute organisms, known as tetanus bacilli.

---

Dr. R. S. Huidekoper will deliver two lectures a month until May next to the Master Horseshoers' Association of New York City.

